# Supplementary material for: Atmospheric-moisture-induced polyacrylate hydrogels for hybrid passive cooling
Source: Nat Commun. 2023 Oct 23;14:6707. doi: 10.1038/s41467-023-42548-0 (PMC10593860; doi:10.1038/s41467-023-42548-0)
Supplement: Supplementary file 1 — Supplementary Information [file 41467_2023_42548_MOESM1_ESM.pdf]

Supplementary Information for

Atmospheric-moisture-induced polyacrylate hydrogels for hybrid passive cooling

Roisul Hasan Galib[1†], Yanpei Tian[2†], Yue Lei[2,3], Saichao Dang[2], Xiaole Li[2,4], Arief Yudhanto[2,4], Gilles Lubineau[2,4] and Qiaoqiang Gan[1,2,*]

[1]Department of Electrical Engineering, The State University of New York at Buffalo, Buffalo, NY 14260, United States

[2]Division of Physical Science and Engineering, King Abdullah University of Science and Technology, Thuwal 23955-6900, Saudi Arabia

[3]School of Architecture and Urban Planning, Chongqing University, Chongqing 400045, China

[4]Mechanics of Composites for Energy and Mobility Laboratory, King Abdullah University of Science and Technology, Thuwal 23955-6900, Saudi Arabia

†These authors contributed equally to this work

*Corresponding author. Email: qiaoqiang.gan@kaust.edu.sa (Q.G.)

Table of Contents:

Supplementary Note 1. Materials and fabrication methods for PAAS photonic film
Supplementary Note 2. Versatile properties demonstrations of PAAS photonic film
Supplementary Note 3. Materials characterizations of PAAS photonic film
Supplementary Note 4. Refractive index extraction and FDTD simulation of PAAS photonic film
Supplementary Note 5. Theoretical model of the radiative and evaporative cooling performance for the PAAS photonic film
Supplementary Note 6. Cooling power characterization of the PAAS photonic film
Supplementary Note 7. Modeling of the energy consumption when buildings are modified by PAAS photonic film

List of Figures and Tables:

Supplementary Figure 1. Experimental setup of the lab-scale fabrication for PAAS photonic film.

Supplementary Figure 2. SEM image of PAAS dry powder.

Supplementary Figure 3. The “green” fabrication process from dry powder to a solid film and the corresponding temperature and humidity variations.

Supplementary Figure 4. Minimum relative humidity for PAAS powders to form a continuous film.

Supplementary Figure 5. Colored PAAS photonic film fabrication.

Supplementary Figure 6. Photos showing that PAAS photonic film is applied on various substrates.

Supplementary Figure 7. Photos demonstrating the red-laser scattering effect of PAAS photonic film.

Supplementary Figure 8. Photos showing the self-healing process of PAAS photonic film in a high-humidity environment.

Supplementary Figure 9. The recycling process of the used PAAS photonic film.

Supplementary Figure 10. Solar reflectance thermal emittance of the pristine and recycled PAAS photonic film.

Supplementary Figure 11. The crosslinking process of adjacent PAAS powders under microscopes.

Supplementary Figure 12. Photos of the environmental chamber.

Supplementary Figure 13. Photos of PAAS photonic film after thermal treatment at various temperatures.

Supplementary Figure 14. Photos of PAAS hydrogel specimens and setup of tensile test.

Supplementary Figure 15. Stress-strain curves of PAAS hydrogel film specimens.

Supplementary Figure 16. Relationship between longitudinal stretch (λ_1) and transverse stretch (λ_2) in PAAS hydrogel film specimens.

Supplementary Figure 17. SEM images of the top surface for PAAS photonic film.

Supplementary Figure 18. SEM image of the PAAS photonic film in a higher magnification ratio.

Supplementary Figure 19. Cross section of the PAAS photonic film under SEM.

Supplementary Figure 20. Optical properties of PAAS photonic film with different water contents.

Supplementary Figure 21. The transmittance of the refitted and original PAAS sample that is sandwiched between two glass slide covers.

Supplementary Figure 22. Reflectance spectra of the dry and wet PAAS photonic film.

Supplementary Figure 23. Transmittance spectrum of PE film over solar and infrared wavelengths.

Supplementary Figure 24. Reflectance spectra of the black shingle and PDMS (T).

Supplementary Figure 25. Optimized thickness of PAAS photonic film for improved cooling performance.

Supplementary Figure 26. Weather data during the outdoor test.

Supplementary Figure 27. Temperature variations of PAAS photonic film, dry PAAS, PDMS (T), and shingle under a clear sky.

Supplementary Figure 28. Hybrid cooling performance of PAAS photonic film under different weather conditions.
Supplementary Figure 29. Validation of continuous water evaporation of PAAS photonic film over the daytime.
Supplementary Figure 30. Heat dissipation enhancement demonstration of PAAS photonic film.
Supplementary Figure 31. Photo of the PAAS photonic film, schematic of a midrise building; The 16 U.S. city's location for cooling energy savings during summer for midrise buildings.
Supplementary Figure 32. Cooling, heating, and total energy consumption for buildings when modified by PAAS photonic film for roof and all surfaces.
Supplementary Figure 33. The average energy saving when the urban area densities vary.
Supplementary Table 1. Optical/thermal properties of PAAS with other reported materials.
Supplementary Table 2. PAAS photonic film unit price calculation.
Supplementary Table 3. Thermal properties of the PAAS photonic film used in energy modeling.
Supplementary Table 4. The 16 U.S. cities assessed for cooling energy savings.

Supplementary Table 1. Comparison of optical/thermal properties of PAAS with other literature. The values marked with an asterisk (*) indicate estimates based on the information provided in the corresponding reference.

**Reference**	**Solar reflectance**	**Thermal emissivity**	**Structure**	**Fabrication method**	**Composition**
Sodium polyacrylate (PAAS)[This work]	0.93	0.99	Single layer	Atmospheric moisture absorption	100% PAAS
Hierarchical Porous Coating (HPC) [1]	0.95	0.98	Single layer	solvent exchange method	Modified Poly(vinylidene fluoride-co-hexafluoropropene) (PVDF) and Cellulose Acetate (CA) polymeric network
poly(N-isopropylacrylamide) (pNIPAm) [2]*	0.92	0.94	Three-part sandwich structure thermal homeostasis (SSTH)	free radical UV-initiated polymerization technique	pNIPAM sandwich
poly (vinyl alcohol) (PVA)–$CaCl_2$ [3]	0.94	0.94	bilayer polymer	freeze-drying/electrostatic-spinning synthesis method	cellulose acetate (CA) network and poly (vinyl alcohol) (PVA)–$CaCl_2$ hydrogel
LiBr-polyacrylamide (PAAm) – poly (vinylidene fluoride-co-hexafluoropropylene) [P(VdF-HFP)] [4]	0.96	0.96	bilayer porous polymer	Photopolymerization and phase inversion method	Li-PAAm hygroscopic hydrogel and poly (vinylidene fluoride-co-hexafluoropropene) porous hydrophobic layer

Supplementary Note 1: Materials and fabrication methods for PAAS photonic film

Moisture-induced scale-up fabrication of PAAS photonic film: The fabrication process of large-scale PAAS photonic film includes three steps: preparing the powder bed, moisturizing dry powder, and drying the fabricated film (Supplementary Figure 1). First, dry PAAS powder is loaded on the feeder, and the feeder's height is adjusted for the desired film thickness. The detailed fabrication process includes: First, the dry PAAS powder from the feeder falls on the bed and the speed of the rolling bed is maintained for uniform distribution of the dry powder. A steam heater is placed over the powder film for spraying moisture at a rate of 20-25 g min^{-1} for 20 mins and the temperature is regulated between 65-70°C. Then, the moisturized film is dried to room temperature using airflow and finally, dry PAAS photonic film is fed into a roller for packaging.

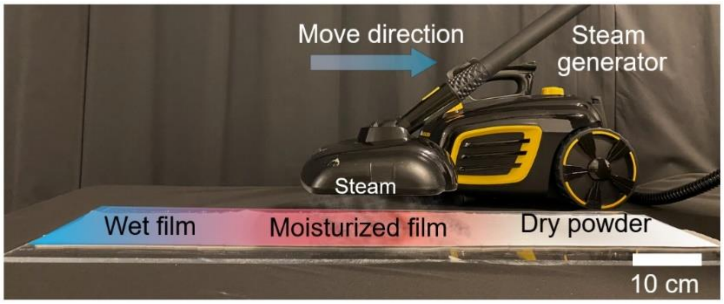

Supplementary Figure 1. Experimental setup of the lab-scale fabrication for PAAS photonic film. The color contour represents stages of PAAS film transformation. The white powder bed on the right is a dry state of PAAS powder. The red portion of the bed in the middle represents the transformation of PAAS powder to PAAS film by absorbing moisture. The blue part on the left indicates the fabricated PAAS film after absorbing moisture.

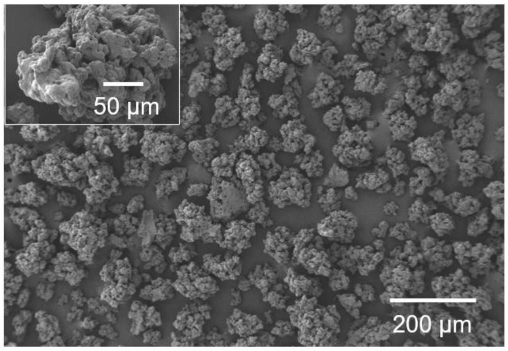

Supplementary Figure 2. SEM image of PAAS dry powder. With an average diameter of ~100 μm, this dry powder is the base of PAAS photonic film fabrication. Starting from the friable power of PAAS, a continuous film is fabricated for cooling purposes.

Atmospheric moisture-induced "green" fabrication of PAAS photonic film: PAAS photonic film is fabricated under ambient-air conditions with a relative humidity of above 60% by absorbing moisture from the ambient environment for 6 hours at night (Supplementary Figure 3). The PAAS particles are activated by water vapor molecules and form a continuous film with uniform density by a hydrogen bond between water molecules. The dry PAAS powders are uniformly distributed on a polyacrylic substrate by the blade coating approach to control its thickness and these powders are placed in an open field at midnight to absorb atmospheric moisture. A corrugated paper box with holes on the sides is employed to protect the sample

from wind or rain and to keep regular air circulation through those holes simultaneously. Different stages of the PAAS from dry powder and moisturized powder to the solid film are listed in Supplementary Figure 3a. The ambient temperature and relative humidity during the fabrication process are shown in Supplementary Figure 3b. PAAS photonic film is formed within 6 hours of absorbing moisture at night. Finally, the fabricated PAAS photonic film can be easily peeled off from the substrate.

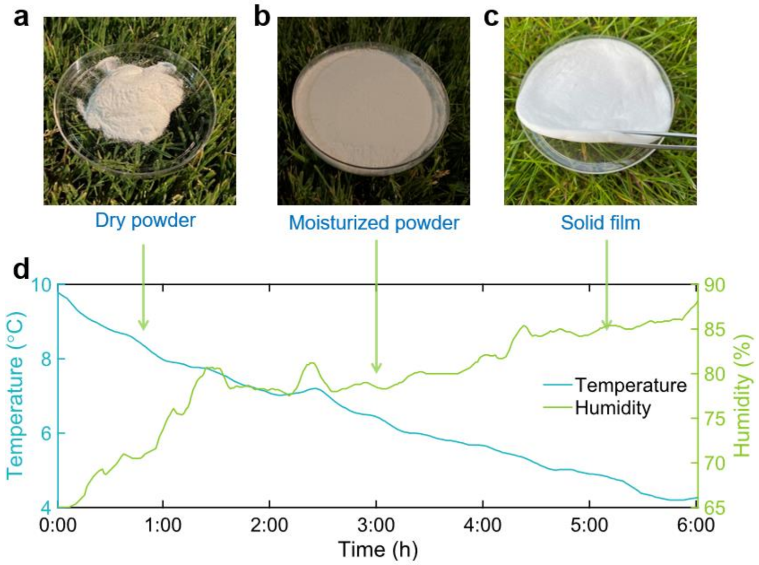

Supplementary Figure 3. The “green” fabrication process of PAAS photonic film from dry powder to a solid film at night and the corresponding temperature and humidity variations. The moisturized powder connects each other and forms a solid white film. The relative humidity ranges from 65% to 90% during the fabrication process and the temperature is from 4°C to 10°C.

Critical relative humidity for PASS powders to form a continuous film: The PAAS film fabrication and regeneration requires a minimum nighttime ambient humidity threshold of 60% RH or higher. To determine this critical threshold, we conducted an experiment involving a PAAS powder sample placed in a controlled environmental chamber set to different relative humidity levels, namely 30%, 50%, 60%, 70% and 90%, all at an ambient temperature of 22℃ for a duration of 6 hours.

After the 6-hour period, we carefully peeled off the PAAS film from the substrate to assess its mechanical stability and film continuity. The findings are presented in Supplementary Figure 4. At 30% RH, no film formation occurred, and the PAAS remained in a powdered state. At 50% RH, PAAS film formation was observed, but the films were discontinuous and exhibited poor mechanical stability. However, from 60% RH and beyond, we noted continuous film formation with satisfactory mechanical stability.

Consequently, based on these observations, we conclusively determined that the threshold for successful PAAS film fabrication and regeneration is 60% RH or higher.

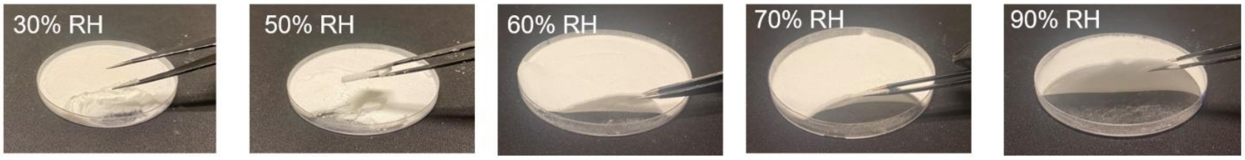

Supplementary Figure 4: Photos showing the continuous film formation of PAAS powders at different relative humidity.

Colorful PAAS photonic film fabrication: To validate the versatility of our proposed atmospheric-moisture-induced fabrication method, we introduce color pigments (Rolio© mica powder) by mixing them with PAAS power powder (1:100 ratio) and then putting them in an environment-controlled chamber (RH, ~ 90%) to form a continuous film with different colors. This procedure is similar to the one described in our manuscript and the only difference is the introduction of pigment powders. The introduced pigment powders did not affect the formation of continuous film, as depicted in Supplementary Figure 5a where four colored PAAS photonic films with different colors are displayed. These films show bright colors to the naked eye which corresponds to the absorption peaks as shown in the spectra results in Supplementary Figure 5b. Moreover, compared with the pristine white PAAS photonic film, these colored PAAS films keep high reflectance over other solar wavelengths (e.g., near-infrared) while displaying unity thermal emittance over infrared wavelengths. The high infrared thermal emittance enables effective radiative heat dissipation through the atmospheric transparent window. This moisture-induced technique for colored photonic film allows for more flexibility in terms of achieving specific color effects or incorporating multiple colors.

Furthermore, a colorful appearance will be more attractive for specific applications, such as wearable electronics, automotive and cooling textiles. However, adding pigment powders to the white radiative cooler may compromise the high solar reflectivity and hence weaken the cooling performance of the film. Therefore, other approaches that can introduce narrowband visible wavelength absorption are highly demanded by further hybrid passive cooling technologies.

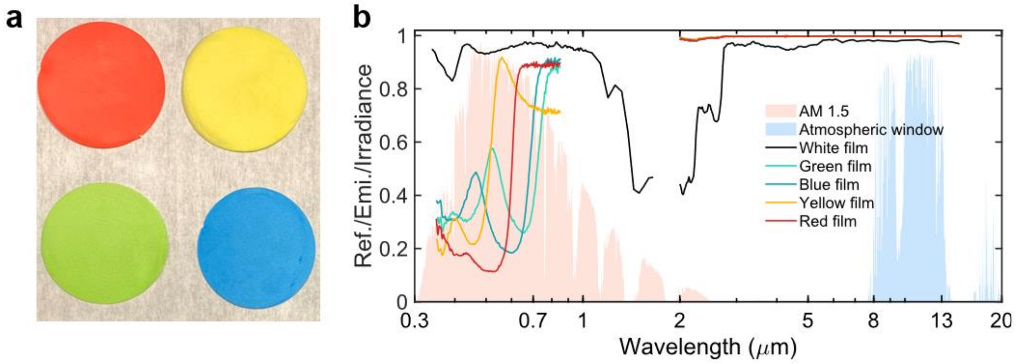

Supplementary Figure 5. (a) Photos displaying the continuous PAAS photonic film of four different colors (red, yellow, green, and blue). (b) Spectra of the colored PAAS photonic film to demonstrate its aesthetic functionality while maintaining effective radiative cooling capabilities.

Supplementary Note 2: Versatile properties demonstrations of PAAS photonic film

Versatile adaption of PAAS photonic film on various surfaces: The PAAS dry power can be applied on different substrates, such as plastic, wood, and metal. After absorbing moisture, these PAAS dry powders will turn into a continuous film and form a bond with the underneath substrate (Supplementary Figure 6). Applying the film on such surfaces requires no adhesives or mechanical pressure. This versatile adaption of PAAS photonic film extends its application in many practical scenarios like the wooden roof of buildings, the metal case of outdoor electronics, and plastics in automotive and various industries.

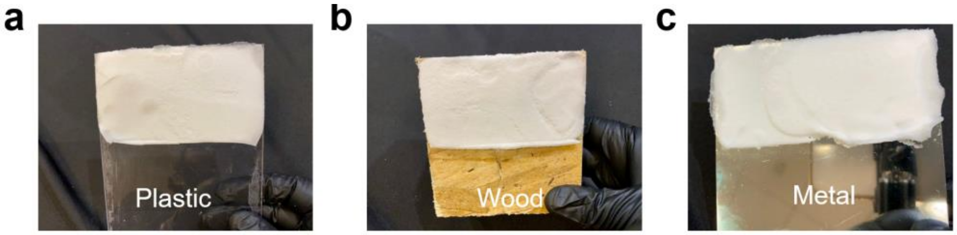

Supplementary Figure 6. Photos showing that PAAS photonic film is applied on various substrates. The PAAS photonic film applied to plastics, wood, and metals significantly modified their surface optical properties. For a transparent plastic board with relatively high thermal emittance, the high solar reflectance of PAAS photonic renders it into an efficient radiative cooler. The yellow-color wood surface is highly absorptive over the visible wavelengths, while the applied white photonic film significantly reduces its solar heating effect and enhances its thermal emittance approaching unity. The metal is highly reflective over thermal infrared wavelengths which drastically limits its cooling potential even though it can reflect certain solar wavelengths. This PAAS photonic film further increases its solar reflectance and also endows it with a diffused appearance that is softer to human eyes. Moreover, the high thermal emittance of PAAS photonic film of 0.99 dramatically engineered the low-emittance metals into an effective radiative cooler.

Scattering effects of the PAAS photonic film: The PAAS photonic film strongly scatters the incident light due to its intrinsic porosity. The scattering feature is demonstrated in Supplementary Figure 7, where a red laser is employed on a regular white paper (Supplementary Figure 7a) and the PAAS photonic film (Supplementary Figure 7b). The laser beam source with a diameter of ~ 2 mm. The PAAS photonic film with a diameter of 3 cm is placed on top of white paper. First, the red laser beam is illuminated on the white paper with a negligible scattering effect, while the shining area of the red laser beam is approaching 3 times that of the white paper.

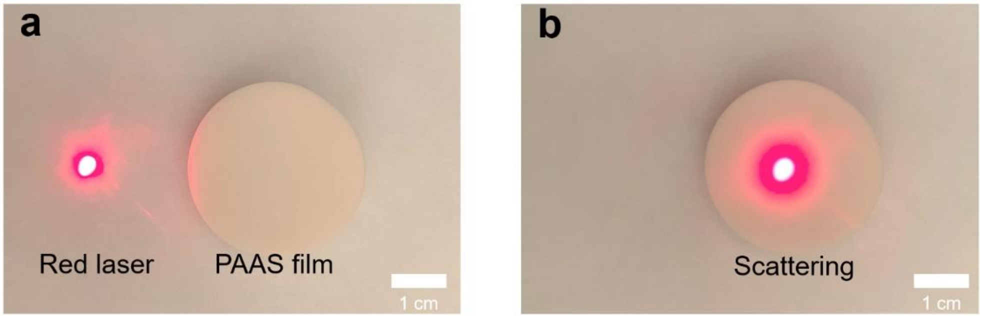

Supplementary Figure 7. Photos demonstrating the red-laser scattering effect of PAAS photonic film. Apart from the backscattering benefits of the PAAS film, the diffused capability of the PAAS photonic film is another advantage compared to the shiny reflective photonic metamaterials which cause twinkling in human eyes.

Self-healing process of the PAAS photonic film: The PAAS photonic film is torn with a slit of ~ 2 cm at its edge. The self-healing process is activated by placing the torn film into a humidity-controlled chamber. The humidity of the chamber is kept at 90% RH for 12 hours. The moisture inside the chamber interacted with the damaged surfaces to form new crosslinks. This bounds the polymer networks together and restores the original polymer's properties. As seen in Supplementary Figure 8, the small slit recovered back to its pristine shape within 12 hours.

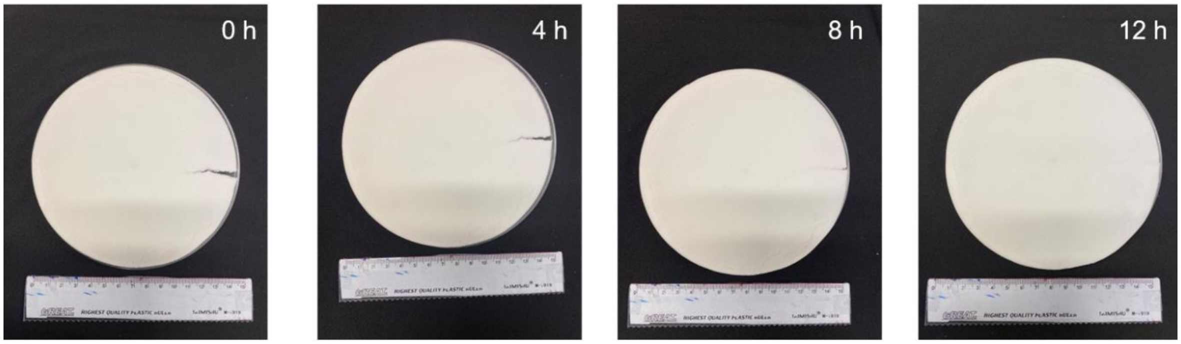

Supplementary Figure 8. Photos showing the self-healing process of PAAS photonic film in a high-humidity environment.

Recycling procedure of the PAAS photonic film: The recycling properties of PAAS photonic film can significantly expand its lifespan and simultaneously increase the carbon downdraw for its overall lifetime. The following photos illustrate its recycling process: The recycling of the PAAS photonic film involves freezing the used film with liquid nitrogen. The extremely low temperature of liquid nitrogen makes the PAAS film very brittle and easy to shatter. Next, the brittle used film is transferred to a blender for crushing it into powders The crushed powder is then mixed with fresh PAAS powder at a ratio of 4:1 (used/fresh) and spread homogeneously on a substrate. Finally, the mixed powder is placed inside a humidity and temperature-controlled chamber (T = ~25°C, RH = 90%) for 6 hours to obtain a continuous film.

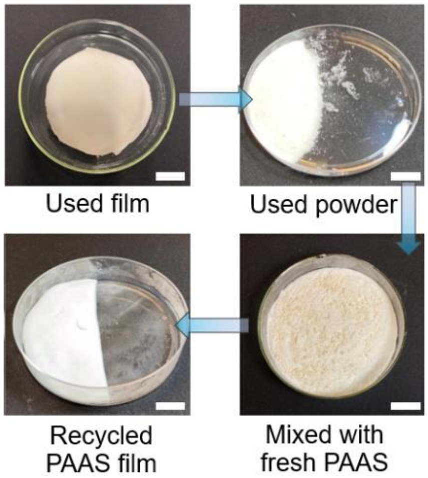

Supplementary Figure 9. The recycling process of the used PAAS photonic film.

The optical performance of recycled PAAS photonic film: The optical performance of the PAAS photonic film is almost identical to the pristine one. The solar reflectance of the recycled PAAS photonic film decreases to 0.945 compared to the pristine one (0.953), while its thermal emittance over 2.5 to 20 μm increases from 0.94 to 0.95.

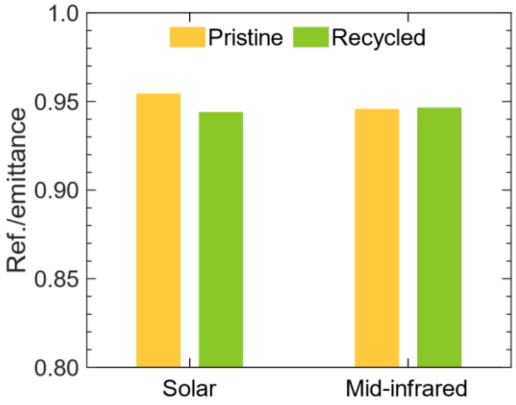

Supplementary Figure 10. Comparison of solar reflectance and thermal emittance of the pristine and recycled PAAS photonic film.

Supplementary Table 2. PAAS photonic film unit price calculation.

Parameter	Unit
PAAS retail price	\$0.99
Sample weight	20 g
Sample area	165 cm^2
Sample thickness	2 mm
Cost	\$1.2 m^{-2}

Supplementary Note 3: Materials characterizations of PAAS photonic film

PAAS film formation mechanism: PAAS film is formed by hydrogen-bond-enabled crosslinking of adjacent powders. In the dry state of PAAS powder, the polymer chain is coiled up in lumped form. When PAAS powders come in contact with moisture, these chains stretch out and start swelling (Supplementary Figure 11). As a result, the molecular chain is extended. This extended chain provides a large number of active points for the water molecules to interact with PAAS. The water molecules rearrange themselves around these active points and interact with ionic groups of PAAS. H^+ ions from water form a bond with carboxylate (COO^-) groups via a hydrogen bond. Since of water's unique structure and polarity, it is capable of associating with two polymer chains. For this reason, water molecules form crosslinks between the negative carboxyl groups (COO^-) of PAAS chains. When adjacent powders connect during swelling, hydrogel bonds form between different PAAS molecules, forming a crosslinked network. Dry PAAS powders are placed on a transparent substrate under an optical microscope. The substrate is placed in a contained and sealed. Moisture is introduced and kept during the whole experiment.

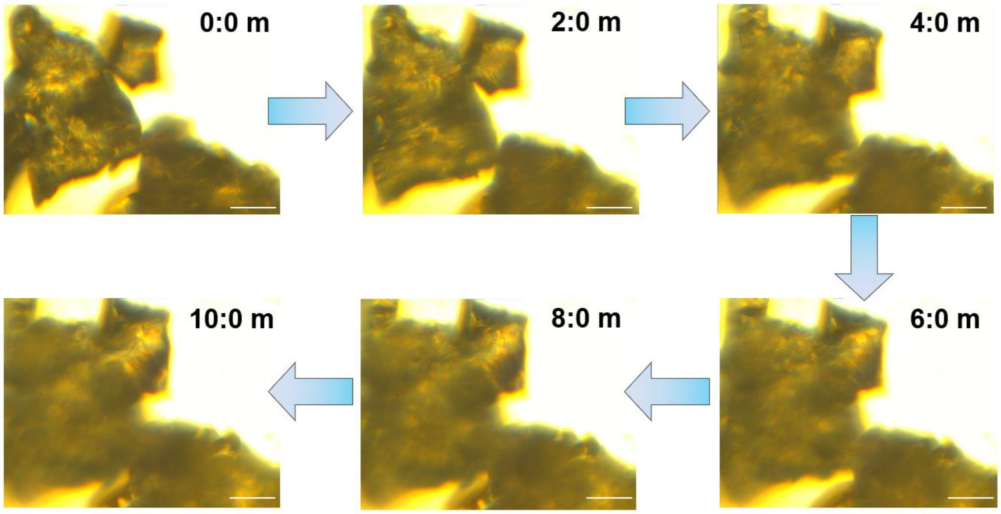

Supplementary Figure 11. The crosslinking process of adjacent PAAS powders under microscopes in 10 minutes. The scale is 25 um. The pictures are taken in 2 minutes intervals.

Experimental setup for controlled humidity chamber: The chamber walls are made of acrylate sheets (Supplementary Figure 12). One sidewall has three ports: moisture is connected to a humidifier, moisture is out, and dry air is connected to a dehumidifier. DI water is used for the humidifier. Anhydrous calcium sulfate (98%) impregnated with cobalt chloride (2%) is used as a desiccant for the dehumidifier. The "ON" and "OFF" of the humidifier and dehumidifier are controlled by a controller to maintain the relative humidity inside the chamber according to our setting. The temperature and relative humidity inside the chamber are recorded by temperature and humidity sensors.

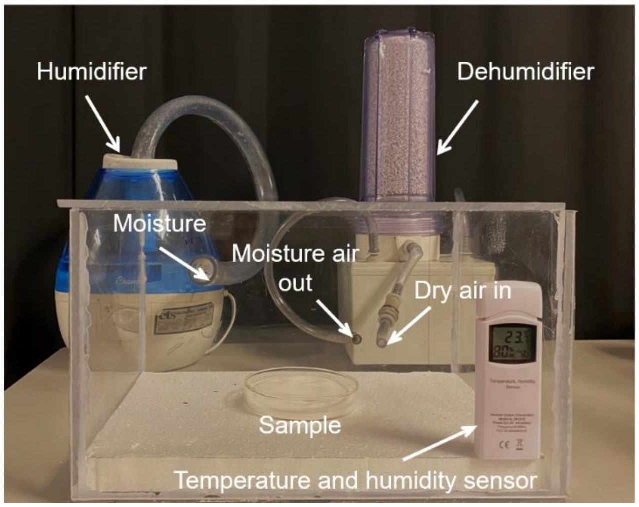

Supplementary Figure 12. Picture of the environmental chamber setup.

Thermal stability of PAAS photonic films: A piece of PAAS film is heated at a predetermined temperature (indicated in Supplementary Figure 13) at 5°C min^{-1}. After reaching the desired temperature, the sample is kept at a constant temperature for 30 mins. Finally, the sample is cooled to room temperature at 10°C min^{-1}. The final state of the sample is shown in Supplementary Figure 11. From the figure, it can be observed that after 200°C the PAAS photonic film would undergo physical and chemical changes visible from its color from white to dark.

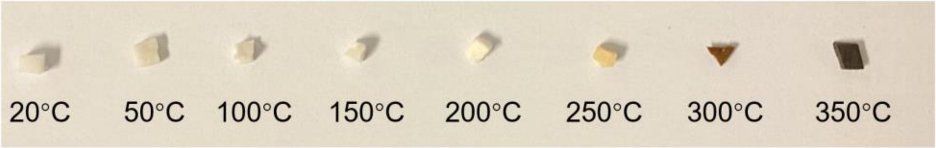

Supplementary Figure 13. Photos of PAAS photonic film color after thermal treatment at various temperatures.

Measurements of mechanical properties of PAAS photonic film: The mechanical properties of sodium polyacrylate (PAAS) photonic film are measured by performing quasi-static tensile tests. The experimental sample and setup are shown in Supplementary Figure 14. Three groups of PAAS hydrogels are prepared (i) Group 1, pristine PAAS photonic film (25 °C, RH, 90%-95%, 6 hours, "pristine"), (ii) Group 2, severed PAAS film and rehealing (25 °C, RH, 90%-95%, 6 hours, "cut and rehealing"), (iii) Group 3, pristine PAAS film and drying (25 °C, RH, 50%-55%, 60 days, "pristine and drying"). The test specimens are prepared by following ISO Standard 527-1BA (see Supplementary Figure 14a) and fabricated by punching the raw materials using a metallic die (Pioneer Dietecs, USA). Supplementary Figure 14b shows the tensile test that is performed using Instron 5944 (2 kN load cell) with a loading speed of 5 mm min^{-1} until the specimen failed, i.e., complete fracture at the gauge length. The load F and displacement data are obtained using *Bluehill 3* software. The stress σ is calculated as follows:

$$\sigma = \frac{F}{wt}$$

where w and t are the initial width and thickness of the specimen, respectively. For the measurement of longitudinal strain (ε_L), we draw two vertical dots on the surface of the specimens and calculated the relative position of these dots using a digital camera. *ImageJ* software is used to track the relative position of two dots, and the strain is calculated by dividing the instantaneous distance between two vertically aligned points at i-th time (u_i) with the initial distance between these two points (u_o) and divided by u_o:

$$\varepsilon_L = \frac{u_i - u_0}{u_0}$$

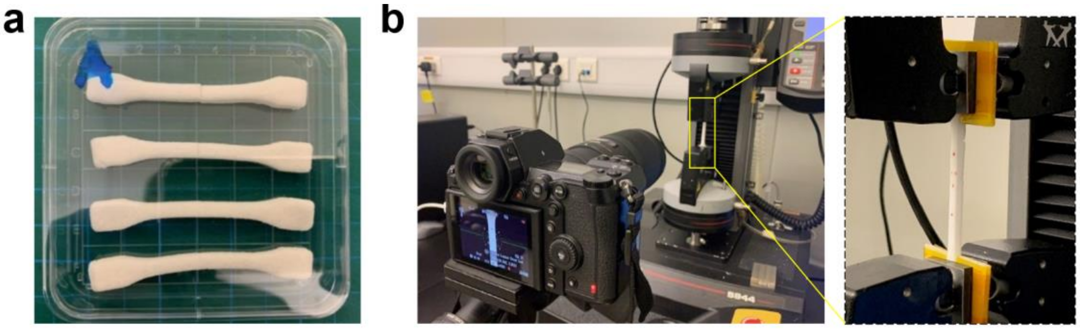

Supplementary Figure 14. (a) PAAS hydrogel specimens. (b) Tensile test setup and PAAS hydrogel specimen under tension.

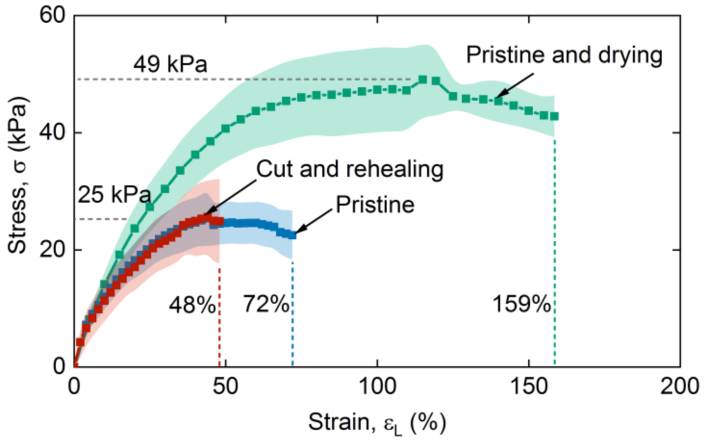

Supplementary Figure 15. Stress-strain curves of PAAS hydrogel film specimens.

Supplementary Figure 15 shows the stress-strain curves of three groups of PAAS photonic film specimens. Group 1: pristine PAAS specimens reach the ultimate stress of 25 kPa and stretchability of around 1.7 times (strain = 72%). Group 2: cut and rehealed PAAS specimens reach the ultimate stress of 25 kPa and stretchability of around 1.5 times (strain = 49%). Group 3: pristine and dried PAAS specimens reach the ultimate stress of 49 kPa with and stretchability of around 2.6 times (strain = 159%) after 60 days. In addition, we also measure the relationship between longitudinal (λ_1) and transverse stretches (λ_1) of the PAAS specimens to show their compressibility. λ_1 and λ_2 can be calculated as follows:

$$\lambda_1 = \frac{u_i}{u_0}$$

$$\lambda_2 = \frac{v_i}{v_0}$$

where v_i is the instantaneous distance between two horizontally aligned points at the *i*-th time, and v_0 is the initial distance between these two points. λ_2 is also calculated using experimental data of λ_1. The "experimental λ_2" is also compared with the "calculated λ_2" (this is generally used for describing an incompressible material):

$$\lambda_2 = \frac{1}{\sqrt{\lambda_1}}$$

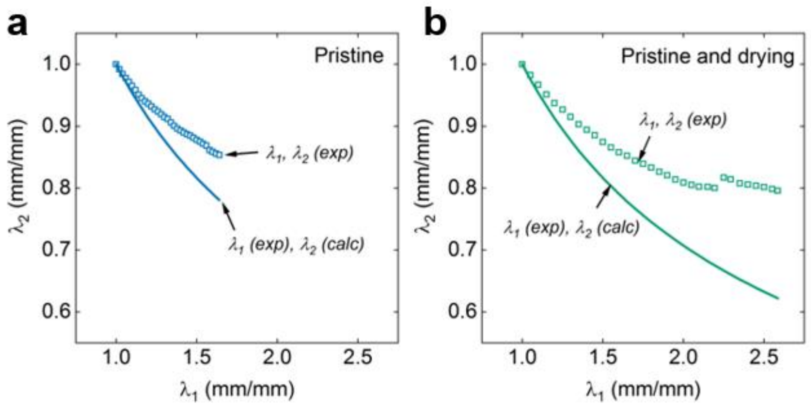

Supplementary Figure 16. Relationship between longitudinal stretch (λ_1) and transverse stretch (λ_2) in PAAS hydrogel film specimens: (a) pristine, (b) pristine and drying.

Supplementary Figure 16 shows that the experimental curves in both “pristine” and “pristine drying” PAAS specimens significantly deviate from the calculated ones, indicating that our PAAS photonic films in either pristine or dried conditions are compressible. This phenomenon is not surprising as the PAAS hydrogel has a foam-like microstructure where its volume could be changed depending on the applied forces. Such compressibility in PAAS photonic film can be explored further for potentially developing a shape-changing or volume-changing material.

Supplementary Note 4: Refractive index extraction and FDTD simulation of PAAS photonic film

SEM characterization of PAAS photonic film: PAAS photonic film's microscopic structure is hierarchical. At a lower magnification ratio, only microstructures are displayed with a size range from 10 μm to 50 μm (Supplementary Figure 17). With the increase of the magnification ratio, nanostructures from 100 nm to 600 nm exist from the top view (Supplementary Figure 18). Supplementary Figure 19 indicates the cross-section of the PAAS photonic films that are similar to its top surface.

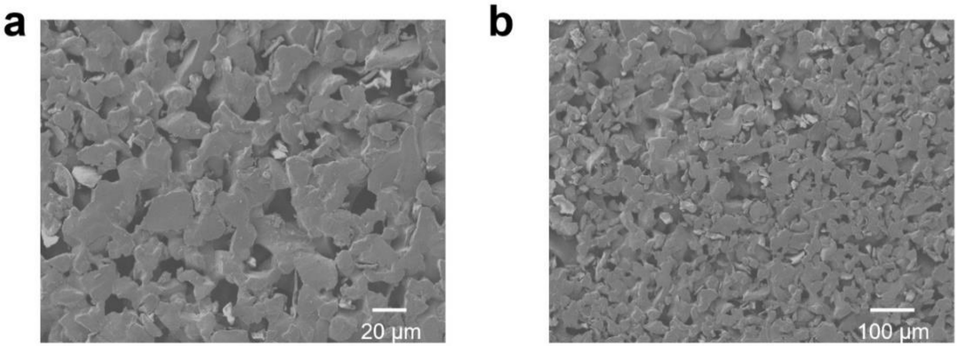

Supplementary Figure 17. SEM images of the top surface for PAAS photonic film.

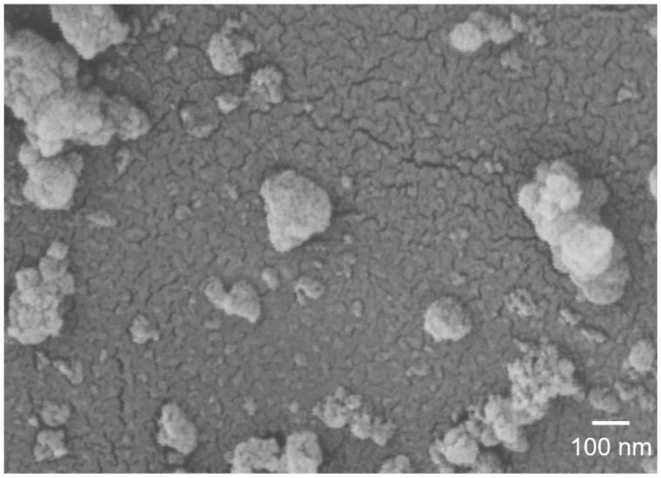

Supplementary Figure 18. SEM image of the PAAS photonic film in a higher magnification ratio.

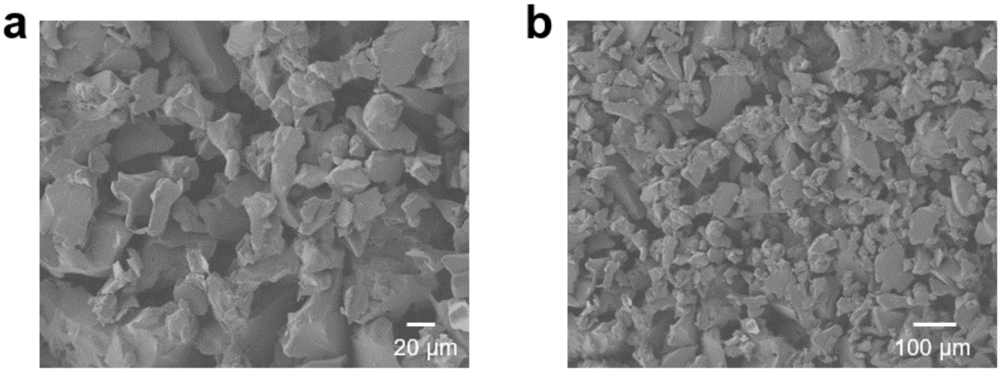

Supplementary Figure 19. Cross section of the PAAS photonic film under SEM.

Variations of optical properties for PAAS photonic film at different water contents: Presented in Supplementary Figure 20 are the changes in optical properties, including reflectance and emissivity, in relation to water vapor. As the water content in the PAAS photonic film increases, a noteworthy effect is observed: the difference in refractive index between PAAS and ambient air diminishes, consequently weakening the backscattering of sunlight. This phenomenon leads to a reduction in reflectance across visible wavelengths. Remarkably, the moisturization of the PAAS photonic film results in a discernible decrease in its scattering effect. It is worth noting that variations in water content do not induce distinguishable differences over infrared wavelengths, as evident in the thermal emittance spectra depicted in Figure R3. These spectra illustrate a remarkable overlap among the curves, indicating negligible changes in the film's behavior in the infrared region for different water contents.

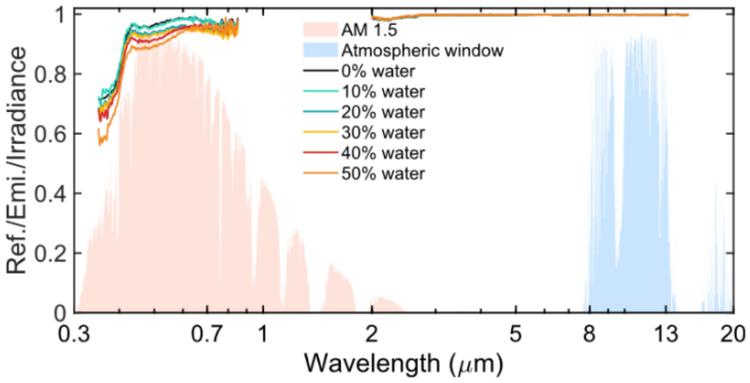

Supplementary Figure 20: Spectra of the PAAS photonic film with different water contents.

Refitting to extract the refractive index of PAAS: The PAAS powders are dissolved into DI water with a weight ratio of 1:500 (PAAS/water) to get a homogeneous solution. Two droplets of PAAS solution are encapsulated between two cover glass slips with a thickness of 0.15 μm for the transmittance spectrum measurement. To estimate the refractive index of PAAS, we use the method outlined by Verleur et al. [5] to extract the refractive index by refitting the transmittance spectrum. Refractive index ($n + i\kappa$) can be assumed to be of Lorentz-Drude oscillator form given by:

$$\epsilon(\omega) = \epsilon_\infty + \sum_{k=1}^{N} \frac{s_k}{1 - \left(\frac{\omega}{\omega_k}\right)^2 - j\Gamma_k(\frac{\omega}{\omega_k})}$$

Here,s_k, ω_k, Γ_k, and j are the strength, resonant frequency, damping factor of *k*th Lorentz-Drude oscillator, and the imaginary unit, respectively. *N* such oscillators are assumed. ϵ_∞ is the contribution from higher frequencies. Since the incidence in the measurement is at 0°, the transmittance spectrum is calculated for that angle of incidence. ω_k correspond to several of the vibration bond resonance of PAAS. Consider a structure having 5-layer media: air, glass, PAAS, glass, and glass, respectively. The refractive index of PAAS is obtained by tunning 5 oscillator parameters by matching the refitted transmittance spectrum to the measured one. This can be done by minimizing the error between the refitted and measured spectra. The minimization is done in two steps: first, we use a MATLAB-based genetic algorithm to arrive at an initial guess of oscillator parameters. This brings us closer to the global optimum of the objective function, providing a reasonable fit that is further improved by using the constrained optimization function *fmincon.* The final refitted spectrum is shown in Supplementary Figure 21. The refractive index is plotted in Figure 3d.

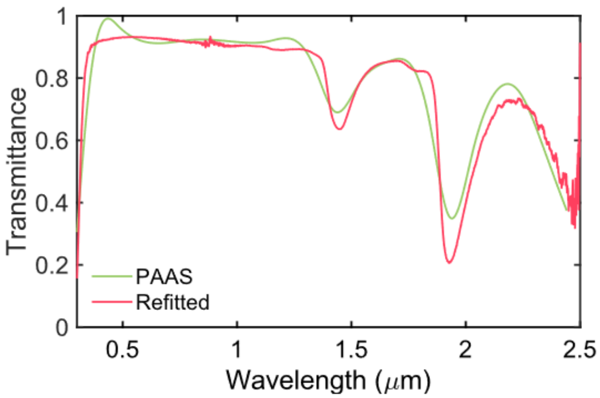

Supplementary Figure 21. Transmittance of refitted and original PAAS sample that is sandwiched between two glass slide covers.

Comparison of reflectance/emittance spectra of dry and moisturized PAAS photonic film: Moisturized PAAS photonic film exhibited reduced solar and IR reflectance/emittance due to the reducing scattering effect (Supplementary Figure 22a-b). When PAAS photonic film is moisturized, the absorbed water in the film causes a reduction in the difference between the refractive indices of PAAS and air. However, thermal emittance in the mid-infrared region is unaffected by water absorption (Supplementary Figure 22c).

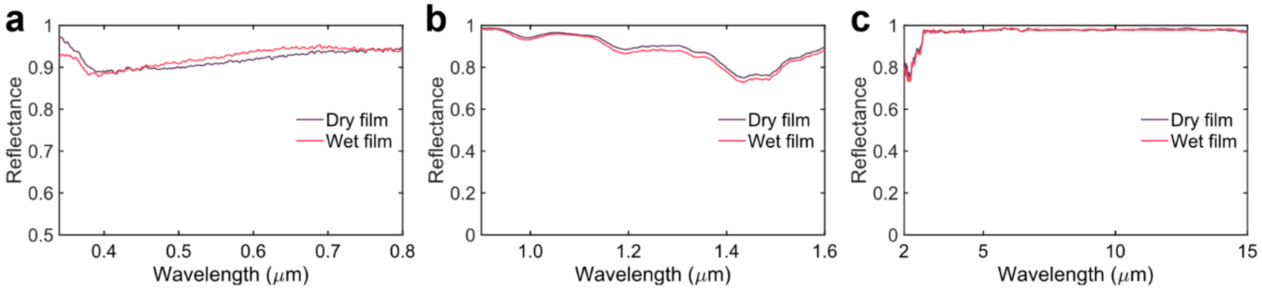

Supplementary Figure 22. Reflectance spectra of the dry and wet PAAS photonic film in (a) visible range, (b) near-infrared region (c) mid-infrared region

Supplementary Note 5: Theoretical model of the radiative and evaporative cooling performance for the PAAS photonic film

The PAAS photonic film supports both radiative and evaporative cooling through water evaporation (P_{vap}), and radiative thermal dissipation (P_{rad}), while reflecting the sunlight to minimize the external solar irradiance (P_{sun}). Meanwhile, it also receives heat from the external atmospheric thermal radiation (P_{atm}), as well as thermal conduction and convection from the ambient environment ($P_{cond+conv}$). Therefore, the net cooling power (P_{net}) can be quantified by:

$$P_{net} = P_{vap} + P_{rad} - P_{sun} - P_{atm} - P_{cond+conv} \quad (1)$$

where $P_{vap} = ER \times \Delta H_{vap}$. ER is the evaporation rate and ΔH_{vap} represents the latent heat of water vaporization (from 2453 to 2429 kJ kg^{-1} corresponding to 20°C and 30°C, respectively). Here, we assume that the radiative cooling power is around 100 W m^{-2} for the radiative heat exchange ($P_{rad} - P_{atm}$) between PAAS film, the atmosphere, and outer space based on the previous experimental work [6-7] and the solar irradiance is 1,000 W m^{-2}. The parasitic heat of convection and conduction ($P_{cond+conv}$) can be calculated by multiplying the combined heat coefficient (h_c) and the temperature difference between the ambient air (T_{amb}) and the PAAS photonic film (T). Therefore, equation (1) can be expressed as:

$$P_{net} = ER \times \Delta H_{vap} + 100\, W\, m^{-2} - 1000(1 - R_{sun}) - h_c(T_a - T) \quad (2)$$

Supplementary Note 6: Cooling power characterization of the PAAS photonic film

Calculation of radiative cooling power: The radiative cooling power of the PAAS photonic film is calculated using the following equation:

$$P_{net} = P_{rad}(T_{\mathrm{PAAS}}) - P_{atm}(T_{amb}) - Psun - P_{nonrad}(T_{\mathrm{PAAS}}, T_{amb}) \quad (3)$$

The radiative power emitted from the PAAS sample is:

$$P_{rad}(T_{\mathrm{PAAS}}) = A\int d\Omega \cos(\theta)\int d\lambda\, I_{BB}(T_{\mathrm{PAAS}}, \lambda)\, \varepsilon_{rad}(\lambda) \quad (4)$$

where $\int d\Omega$ is the angular integral of the emitting surface over the hemisphere, $I_{BB}(T, \lambda) = 2hc^2/\lambda^5$ (1-exp($h_c/\lambda k_B T$)−1) is the spectral radiance of a blackbody at a temperature T, h is Planck's constant, k_B is the Boltzmann constant, c is the speed of light, λ is the wavelength, and A is the area of the emitter. ε_{rad} is the spectral emissivity of the PAAS photonic film. The emissivity of the PAAS photonic film is obtained from **Figure 1d**.

The absorbed atmospheric radiation from the emitter is:

$$P_{atm}(T_{amb}) = A\int d\Omega \cos(\theta)\int d\lambda\, I_{BB}(T_{amb}, \lambda)\, \varepsilon_{rad}(\lambda)\, \varepsilon_{\mathrm{atm}}(\theta, \lambda) \quad (5)$$

Here, the angle-dependent emissivity of atmospheric $\varepsilon_{\mathrm{atm}}(\theta, \lambda)$ is given by $\varepsilon_{atm}(\gamma) = 1 - [t_{atm}(0)]1/\cos\gamma$, where the atmospheric transmittance in zenith direction $t_{atm}(0)$ is estimated by $t_{atm}(0) = 1 - \varepsilon_{atm}(0)$. In this estimation, the atmospheric emittance is obtained from MODTRAN (using the atmosphere model: mid-latitude summer).

The absorbed solar irradiation is $P_{sun} = A\int d\lambda\, I_{AM1.5}(\lambda)\, \varepsilon_{rad}(\lambda)$. Here $I_{AM1.5}(\lambda)$ is the AM1.5 Global tilt solar illumination with an irradiance of 1000 W m^{-2}. The solar irradiance is obtained from national renewable energy laboratory.

The non-radiative heat loss due to heat conduction and convection is:

$$P_{nonrad}(T_{rad}, T_{amb}) = A\, h_c(T_{rad} - T_{amb}) \quad (6)$$

Where h_c is the combined heat transfer coefficient, T_{amb} is the ambient temperature, and T_{rad} is the surface temperature of the PAAS photonic film. The value of h_c is determined by:

$$h_c = 2.8 + 3\, v_a$$

where v_a is the velocity of the wind in m s^{-1}. The value of v_a is obtained from weather data at the time of the experiment.

Using these equations, the net cooling powers is calculated, as shown in **Figure 4b**.

Optical transmission properties of PE film: The PE film with a thickness of ~ 15 μm is transparent (> 0.9) over both solar and infrared wavelengths.

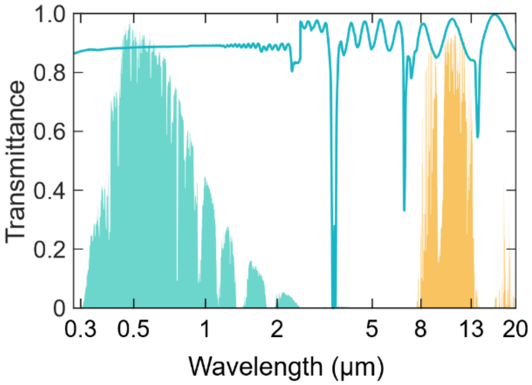

Supplementary Figure 23. Transmittance spectrum of PE film over solar and infrared wavelengths.

Reflectance spectra of black shingle and transparent PDMS:

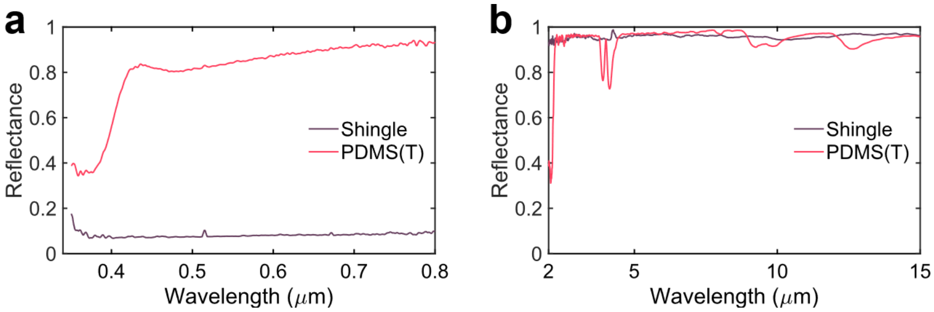

Supplementary Figure 24. Reflectance spectra of the black shingle and PDMS (T). Even though the PDMS (T) and shingle show high thermal emittance over thermal emittance range, the solar reflectance of PDMS (T) is 0.83 that is lower than the PAAS photonic film. Even worse, the black shingle with a solar absorptance of 0.95 is an efficient solar absorber.

Optimized thickness of PAAS photonic film for improved cooling performance: The thickness of the PAAS film plays a pivotal role in determining the overall performance of the cooling system, and it comes with its own set of advantages and disadvantages.

One notable disadvantage of thicker films is their potential to hinder the effective emission of thermal radiation from the underlying radiative cooling films. This could result in a decrease in the overall radiative cooling capacity, especially if the film absorbs and scatters a significant amount of the outgoing thermal radiation. However, there are several significant advantages associated with a thicker PAAS film:

The primary advantage is the increased moisture absorption capacity enabled by the greater thickness. A thicker film can store a larger volume of water (as demonstrated in Supplementary Figure 24a), facilitating prolonged and continuous evaporative cooling. This attribute proves especially valuable in arid or semi-arid climates, where water availability may be limited. Additionally, thicker PAAS films have a higher heat capacity, allowing them to store more thermal energy during the day. Consequently, the cooling effect can be sustained for extended periods, even after sunset. Moreover, increased thickness often imparts mechanical robustness and durability to the PAAS film, rendering it more resistant to physical damage and wear. This enhanced durability contributes to extending the operational lifespan of the cooling application.

In response to the impact of the PAAS film's thickness on its performance, we conducted an additional outdoor experiment in this revision. This experiment involved testing PAAS films with varying thicknesses to assess their cooling performance, and we carefully monitored the solar intensity and relative humidity during the experimental period (as displayed in Supplementary Figure 24b). Our findings indicated that while a thicker hydrogel film indeed enhances moisture absorption and heat storage capabilities, it also introduces certain trade-offs, such as reduced radiative cooling efficiency. After careful evaluation, we determined that a 2-mm-thick film provided the optimal cooling performance (as shown in the temperature response graph in Supplementary Figure 24c). The PAAS photonic film with a thickness of 2 mm exhibited the lowest temperature during the experimental period. This particular thickness strikes a balance between moisture absorption capacity and radiative cooling efficiency, ultimately delivering the best overall cooling performance. Consequently, this thickness value is employed and discussed in our manuscript.

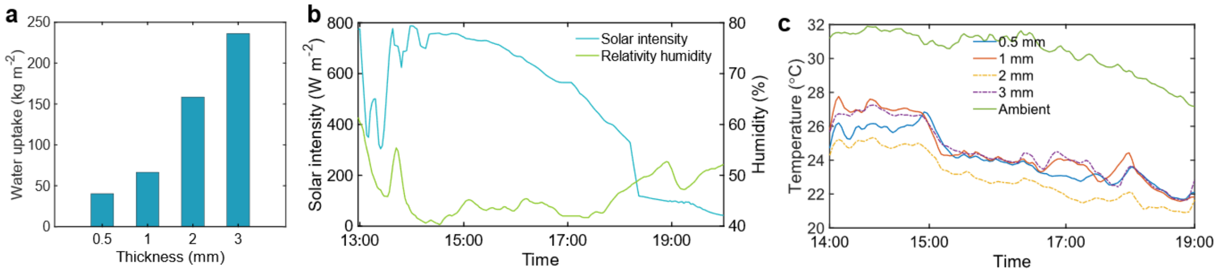

Supplementary Figure 25**:** (a) Water uptake of PAAS photonic film at different thicknesses. (b) The solar intensity and relative humidity during the experimental period. (c) Temperature variation of the hybrid and radiative cooling samples during the experiment.

Weather data (relative humidity) during outdoor test:

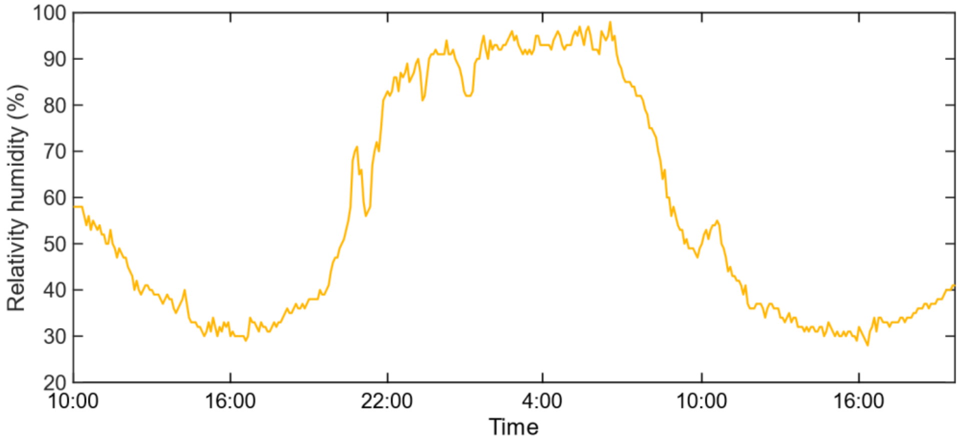

Supplementary Figure 26. Relative humidity variation for the one-day cycle of the outdoor cooling performance measurement.

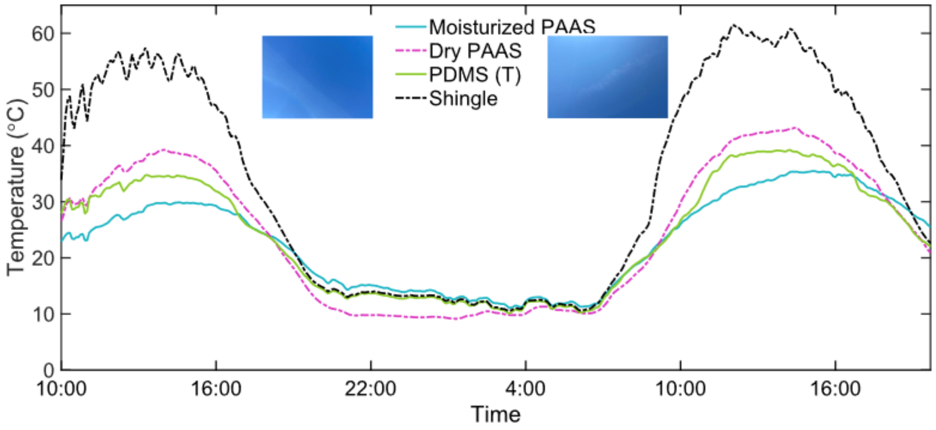

Supplementary Figure 27. Temperature variations of PAAS photonic film, dry PAAS, PDMS (T), and shingle under a clear sky. Insets are pictures of a clear sky during noon time.

Hybrid cooling performance of PAAS photonic film under different weather conditions: To further demonstrate the superior cooling performance of PAAS photonic film, we tested our materials for longer periods of time under various climatic and boundary conditions. Specifically, we performed uninterrupted tests for three consecutive days with different weather conditions, as shown in Supplementary Figure 28.

On Day 1, we tested our material in a sunny sky with smoky air, due to suspended pollutant particles (PM2.5 average particle size 2.5 micron) from wildfires, which led to unhealthy air quality (AQI 150+) and scattering of PAAS photonic film emissions to the sky. Consequently, the radiative cooling effect was hindered, and the radiative cooler only reached around ambient temperature. However, the hybrid cooler exhibited a mean 4°C subambient cooling at noontime, due to its evaporative cooling functionality, which remained insensitive to this extreme weather condition (see day 1 result in Supplementary Figure 28a).

On Day 2, we tested the material under a partly cloudy sky and hot day. The mean solar intensity was ~ 850 W m^{-2} (see day 2 result in Supplementary Figure 28b) and the average ambient temperature was 32°C. Even under such conditions, the hybrid cooling material realized a subambient cooling of 8°C under peak solar intensity, outperforming the radiative cooling material by 4°C.

Lastly, on Day 3, we measured the cooling performance on a mostly cloudy where radiative heat transfer is blocked to the universe. As expected, the radiative cooler struggled to achieve sub-ambient cooling, reaching the ambient temperature at noontime. However, the hybrid cooler still maintained a temperature ~5°C below ambient, highlighting its capability to perform well under different extreme weather conditions. Our results show the hybrid cooler performs well under different weather conditions (see day 3 result in Supplementary Figure 28b).

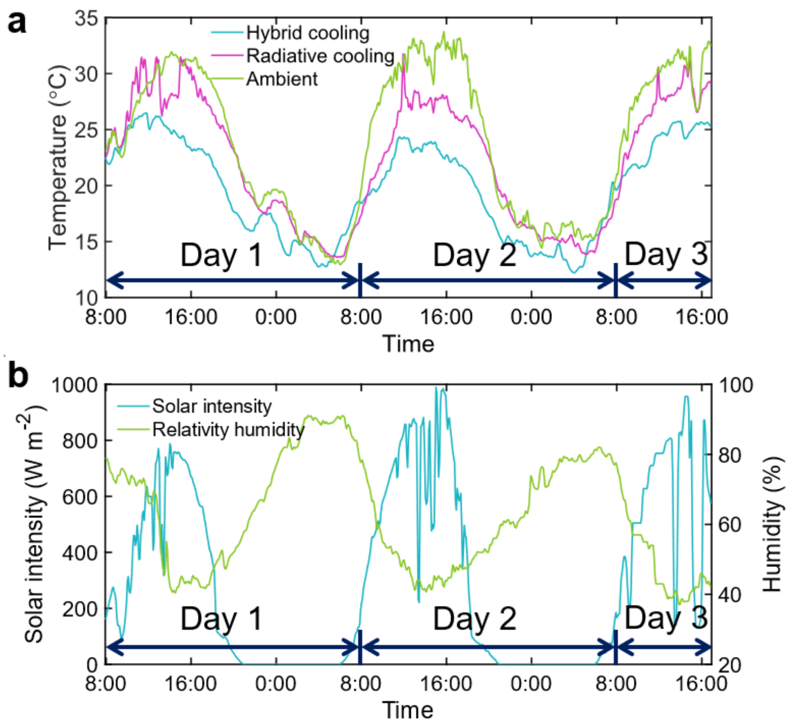

Supplementary Figure 28: (a) Temperature variation of the PAAS photonic film under two scenarios: hybrid cooling and radiative cooling. (b) The corresponding solar intensity and humidity during the 3-day experimental period.

Validation of continuous water evaporation of PAAS photonic film over the daytime: The material's effective working duration is notably influenced by prevailing weather conditions. In order to demonstrate its exceptional cooling ability, we conducted a rigorous experiment on July 12, 2023, specifically focusing on measuring the temperature reduction achieved solely through evaporative cooling. This experiment was carried out in Buffalo, NY. During the experiment, we observed that the PAAS photonic film exhibited remarkable evaporative cooling performance for a minimum of 9 hours and possibly even longer. To accurately gauge the cooling effect, we devised an experimental setup closely resembling the outdoor test described in our manuscript. The only deviation was the use of an aluminum cover, which effectively blocked access to the sky and thereby prevented radiative cooling. However, to facilitate the escape of vapor for evaporative cooling, we left a gap between the aluminum cover and the sample.

The temperature response of the PAAS photonic film was carefully recorded and is presented in Supplementary Figure 29a. Notably, throughout the duration of the experiment, the film's temperature consistently remained below the ambient temperature, unequivocally validating the occurrence of evaporative cooling from approximately 11 a.m. to 9 p.m. Additionally, we monitored the water content of the PAAS photonic film during the experiment. As depicted in Supplementary Figure 29b, the water content steadily decreased from 0.7 g/g to nearly zero, demonstrating its consistent ability to sustain water evaporation throughout the day. This reliable evaporation process effectively maintained a temperature reduction from the ambient during the daytime, when the demand for cooling is more significant than at nighttime. For a comprehensive understanding of the experimental conditions, we also illustrated the variations in relative humidity and solar intensity over the entire experimental period in Supplementary Figure 29c. These details provide essential context and further support the validity of our findings.

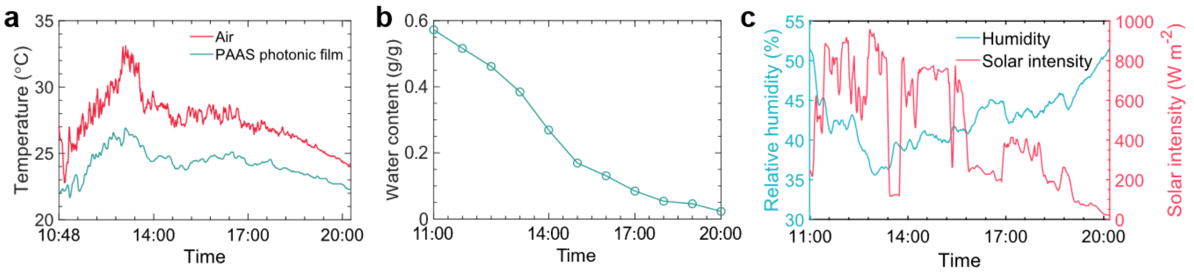

Supplementary Figure 29: (a) Temperature response of the PAAS photonic film when only evaporative cooling happens. (b) Water content changes over the daytime of the PAAS photonic film. (c) Relative humidity and solar intensity variation over the experimental period.

Heat dissipation enhancement demonstration of PAAS photonic film: The combination of hybrid cooling mechanisms can lead to distinct cooling effects in enclosures with a heat source inside. Heat transfer in open areas mainly occurs by conduction and convection, but the mechanism is quite different in an enclosed space with heat accumulating in enclosed space, such as outdoor electronics and parked vehicles. In order to validate this effect, we conducted temperature recording experiments covering an aluminum enclosure with PAAS photonic film under clear sky conditions, which simulates a case of outdoor electronics (Supplementary Figure 30a). We place a temperature probe inside three hollow enclosures and use three different covers: (1) bare enclosure, (2) enclosure with radiative PAAS film, and (3) enclosure with hybrid PAAS film. The heater was powered by a DC power supply set at a voltage of 3.0 V and a current of 0.25 A, resulting in a heat flux of 300 W m^{-2} to simulate the heat generation of electronic devices. The results showed bare enclosure temperature is 9°C above ambient. However, when the top enclosure is covered with PAAS films, both the hybrid and radiative PAAS films exhibited comparable sub-ambient performance. Notably, the hybrid PAAS film demonstrated a significant temperature reduction of 6.1°C compared to the bare enclosure (Supplementary Figure 30b). This outcome indicates that the PAAS film effectively releases trapped heat and blocks solar radiation from entering the enclosure. The combination of evaporation and radiative cooling, as demonstrated by our hybrid PAAS film, indeed presents a unique and enhanced cooling behavior that can be advantageous in various applications. By integrating both cooling mechanisms, the system can achieve efficient cooling even under challenging environmental conditions, such as in high-temperature or high-humidity environments.

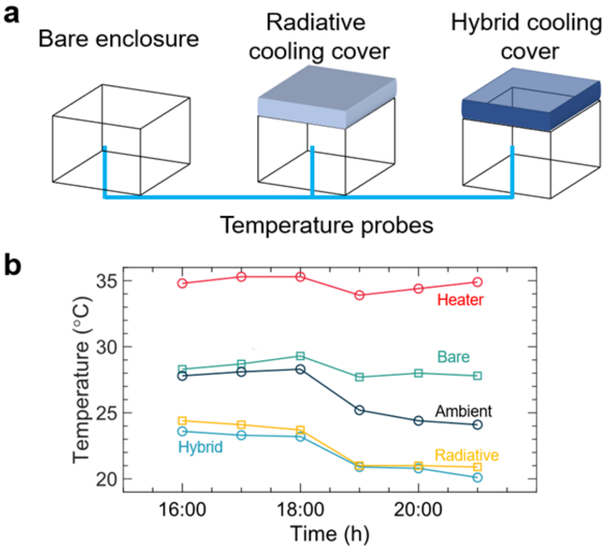

Supplementary Figure 30: (a) Schematics showing the experimental setup. (b) Temperature response of the bar Al enclosure, and with radiative cooling or hybrid cooling PAAS photonic film.

Supplementary Note 7: Modeling of the energy consumption when buildings are modified by PAAS photonic film

EnergyPlus simulation is performed to demonstrate the potential energy including heating, cooling, and total energy consumption by installing PAAS photonic film on post-1980 mid-rise apartment building exterior roof membrane including (1) the exterior wall siding and (2) the exterior roof. The midrise apartment buildings are used from the DOE Reference Buildings database to establish an energy use baseline [8]. The modified cases use PAAS film in place of the roofing and wall material, with the material properties obtained in laboratory experiments as specified in Supplementary Table 3. We consider the most common type of residential building medium-sized midrise apartments located in 16 geographic locations (Supplementary Figure 24). We calculated the cooling energy saving enabled by PAAS photonic film in all 16 climate zones in the United States (Supplementary Table 4) based on the Typical Meteorological Year 3 (TMY3) hourly weather data [9]. Building energy models account for a total heat balance on both internal and external building enclosure surfaces, heat transfer through the building enclosure, and heat sources/sinks, such as internal loads generated by equipment, occupants, and lighting. These models use ray tracing for all components of radiative heat transfer, including direct and indirect fluxes, and fluxes reflected from both the ground and surrounding building surfaces.

Supplementary Figure 25 shows the detailed energy analysis of baseline and modified energy consumption patterns when the roof and wall are modified by PAAS photonic film. Among these cities, Miami (24.63 GJ), Houston (16.98 GJ), Phoenix (16.03 GJ), Atlanta (14.74 GJ), and Los Angeles (11.64 GJ) featured net cooling energy savings when only the roof is modified. Even more energy-saving yields are demonstrated when both roof and wall are modified by PAAS photonic film, i.e., Miami (81.78 GJ), Houston (60.62 GJ), Phoenix (104.14 GJ), Atlanta (46.13 GJ), Los Angeles (51.58 GJ). For other cities, the heating energy offsets the potential cooling gains, and the total energy consumption is increased after buildings' external surfaces are modified. Therefore, the installation of PAAS photonic film for midrise apartment buildings is favorable for cities with warm/hot climates with long and hot summers and short and warm winters.

Supplementary Figure 26 provides the average energy consumption of different area building densities when only the roof or both roof and walls are modified by PAAS film. The urban densities with distances between buildings of 3, 9, and 15 meters are set according to the suggested distances reported in the Lot and Building standards. Therefore, these four different urban area densities represent realistic urban conditions to study the effect of radiation reflected from neighboring buildings as well as the shading effect they provide to the building covered with PAAS photonic film. When only the roof is modified by PAAS film (Supplementary Figure 26a), the average energy consumption reduces when cities are located in hot/warm climates, while the energy consumption increases, e.g., Miami (560 MJ m^{-2}-isolated; 498 MJ m^{-2}-15 m; 493 MJ m^{-2}-9 m; 484 MJ m^{-2}-3 m) and Fairbanks (1467 MJ m^{-2}-isolated; 1489 MJ m^{-2}-15 m; 1501

MJ m^{-2}-9 m; 1512 MJ m^{-2}-3 m). This is because the surrounding buildings decrease the cooling gains due to the limited sky view factors when building densities increases. The radiated energy from the roof is absorbed by the surrounding buildings surfaces. But for cities in cold climates, the potential cooling savings associated with the PAAS photonic film is reversed during the winter months by the huge increase in heating energy. Additionally, the shading effect of neighboring buildings will increase the heating energy consumption in the building of interest. This means that the average energy consumption for cities in cold climate increases when the area density increases.

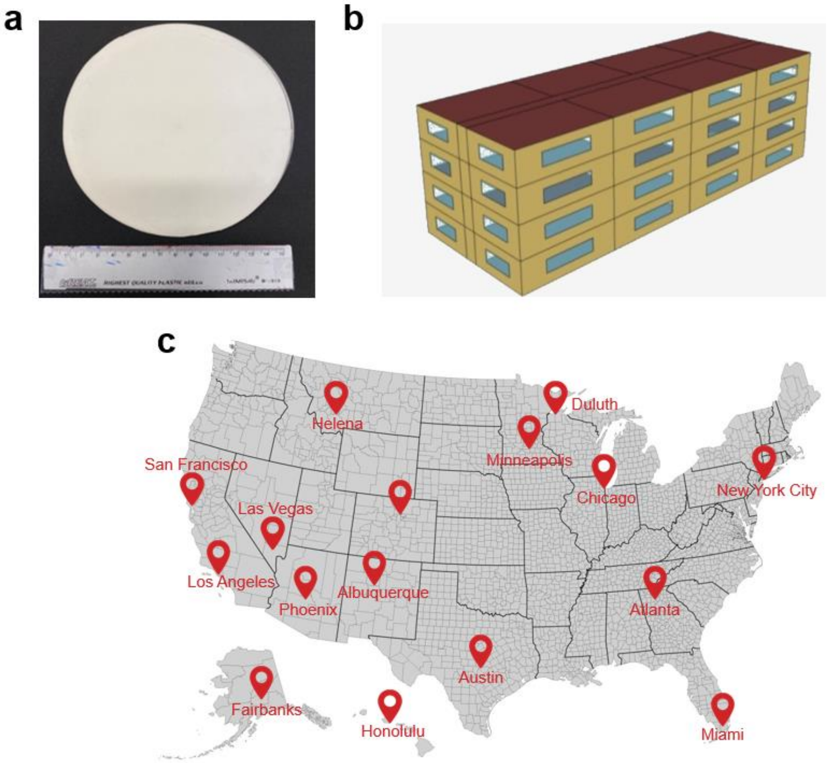

Supplementary Figure 31. (a) Photo of the PAAS photonic film. (b) Schematic of a midrise building. (c) The 16 U.S. city's location for cooling energy savings during summer for midrise buildings.

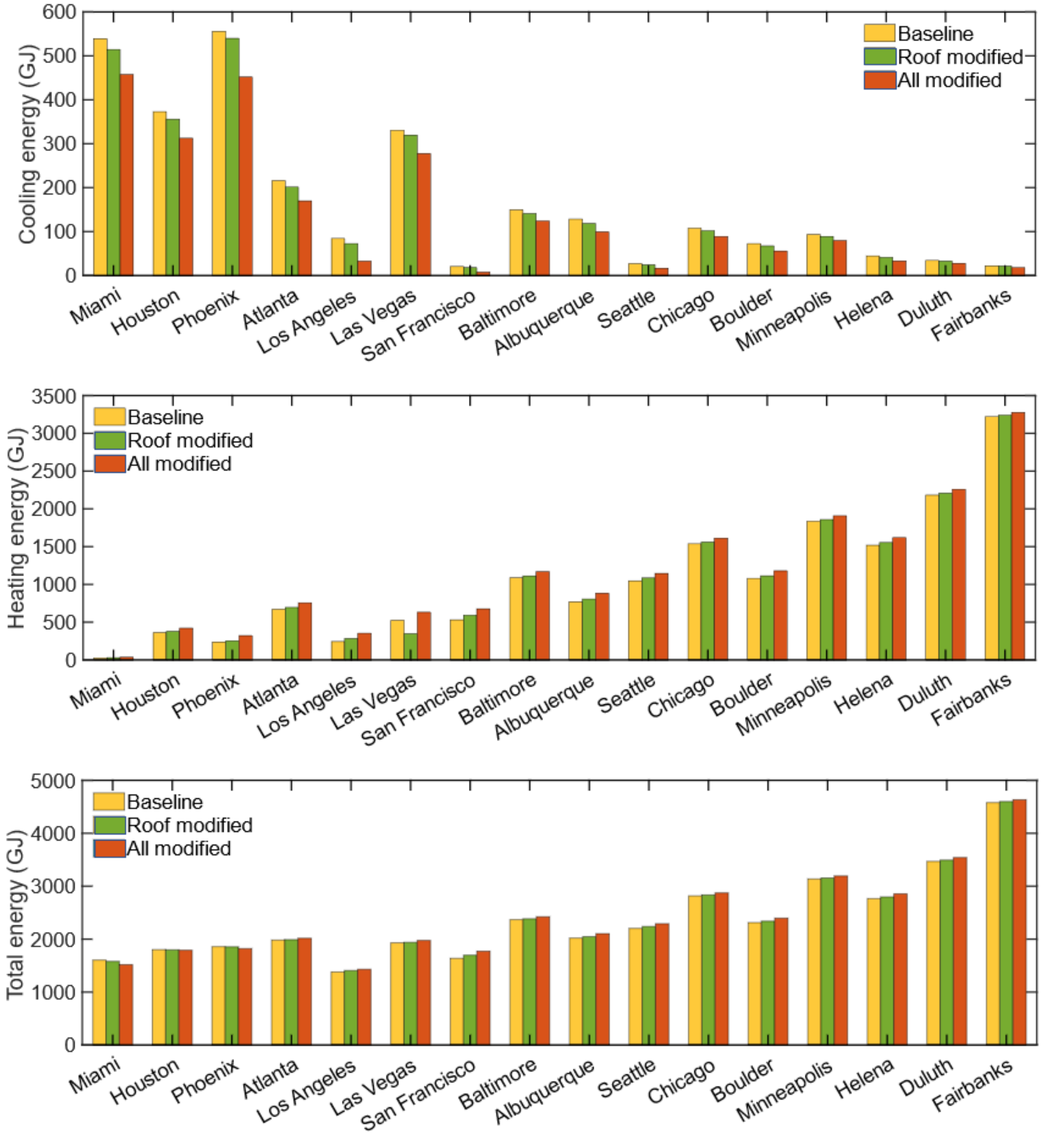

Supplementary Figure 32. Cooling, heating, and total energy consumption for buildings when modified by PAAS photonic film for roof and all surfaces.

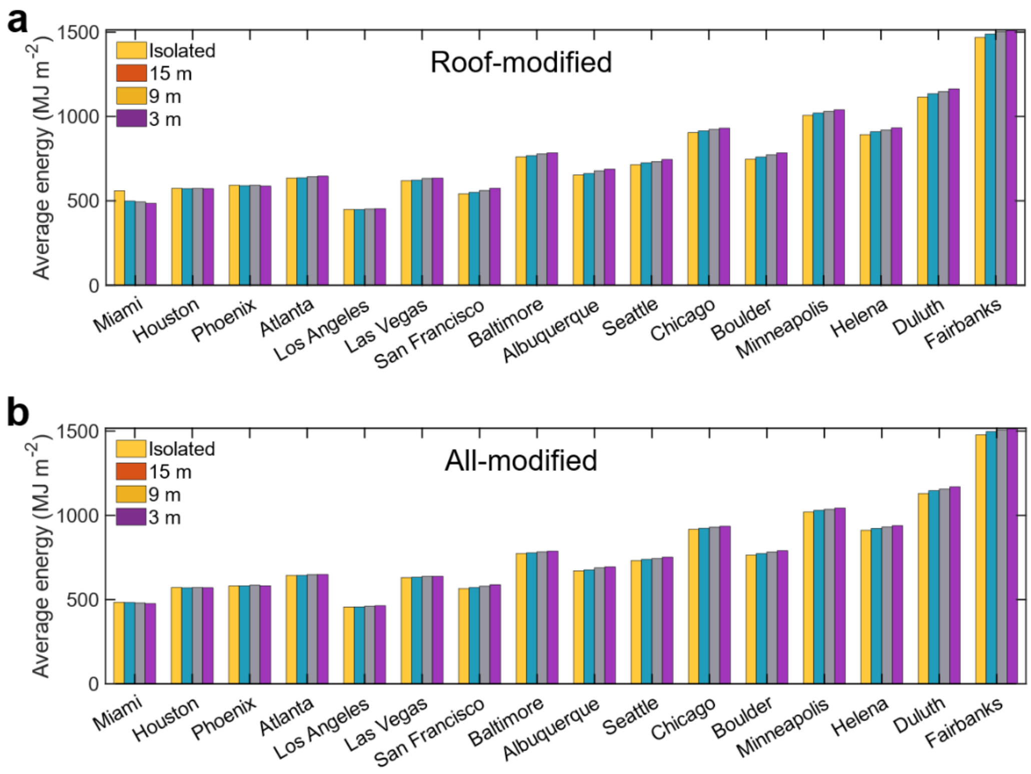

Supplementary Figure 33. The average energy saving when the urban area densities vary.

Supplementary Table 3. Thermal properties of the PAAS photonic film used in the energy modeling.

**Property**	**Value**
Thickness (mm)	3
Specific heat capacity (kJ $kg^{-1}K^{-1}$)	1940
Thermal absorptance	0.9
Solar absorptance	0.1
Visible absorptance	0.1
Thermal conductivity (W m^{-1} K^{-1})	0.25
Density (kg m^{-3})	1320

Supplementary Table 4. The 16 U.S. cities assessed for cooling energy savings.

City, State	Climate	Zone
Miami, Florida	Hot-Humid	1A
Houston, Texas	Hot-Humid	2A
Pheonix, Arizona	Hot-Dry	2B
Atlanta, Georgia	Mixed-Humid	3A
Los Angeles, California	Hot-Dry	3B
Las Vegas, Nevada	Hot-Dry	3B
San Francisco, California	Marine	3C
Baltimore, Maryland	Mixed-Humid	4A
Albuquerque, New Mexico	Mixed-Dry	4B
Seattle, Ishington	Marine	4C
Chicago, Illinois	Cold	5A
Boulder, Colorado	Cold	5B
Minneapolis, Minnesota	Cold	6A
Helena, Montana	Cold	6B
Duluth, Minnesota	Very Cold	7
Fairbanks, Alaska	Subarctic	8

Supplementary References

1. Fei, J., Han, D., Ge, J., Wang, X., Koh, S. W., Gao, S., ... & Li, H. (2022). Switchable surface coating for bifunctional passive radiative cooling and solar heating. Advanced Functional Materials, 32(27), 2203582.
2. Fang, Zhen, Liyun Ding, Lintao Li, Kun Shuai, Boyu Cao, Yetao Zhong, Zhenghua Meng, and Zhilin Xia. "Thermal homeostasis enabled by dynamically regulating the passive radiative cooling and solar heating based on a thermochromic hydrogel." ACS Photonics 8, no. 9 (2021): 2781-2790.
3. Li J, Wang X, Liang D, Xu N, Zhu B, Li W, Yao P, Jiang Y, Min X, Huang Z, Zhu S, Fan S, Zhu J. A tandem radiative/evaporative cooler for weather-insensitive and high-performance daytime passive cooling. Sci Adv. 2022 Aug 12;8(32)
4. Feng, Chunzao, Peihua Yang, Huidong Liu, Mingran Mao, Yipu Liu, Tong Xue, Jia Fu et al. "Bilayer porous polymer for efficient passive building cooling." Nano Energy 85 (2021): 105971.
5. Verleur, H. W., (1968). Determination of optical constants from reflectance or transmittance measurements on bulk crystals or thin films. JOSA, 58(10), pp.1356-1364.
6. National Renewable Energy Laboratory. (2008). National Solar Radiation Data Base: 1991–2005 Update: Typical Meteorological Year 3.
7. Li, T., Zhai, Y., He, S., Gan, W., Wei, Z., Heidarinejad, M., ... & Hu, L. (2019). A radiative cooling structural material. Science, 364(6442), 760-763.
8. Zhai, Y., Ma, Y., David, S. N., Zhao, D., Lou, R., Tan, G., ... & Yin, X. (2017). Scalable-manufactured randomized glass-polymer hybrid metamaterial for daytime radiative cooling. Science, 355(6329), 1062-1066.
9. Zeng, S., Pian, S., Su, M., Wang, Z., Wu, M., Liu, X., ... & Tao, G. (2021). Hierarchical-morphology metafabric for scalable passive daytime radiative cooling. Science, 373(6555), 692-696.
